# Supplementary material for: Hedgehog regulation of epithelial cell state and morphogenesis in the larynx
Source: eLife. 2022 Nov 18;11:e77055. doi: 10.7554/eLife.77055 (PMC9718526; doi:10.7554/eLife.77055)
Supplement: Supplementary file 1. — Cell numbers were quantified from the ventral half of the epithelium in each section analyzed. This table provides source data for figure panels Figure 4K–L and Figure 4—figure supplement 4A–F. Ventral epi. – ventral epithelium. [file elife-77055-supp1.docx]

**Supplementary File 1**

| Embryonic stage | Somite | Genotype | DAPI+ cells (ventral epi) | TdT+cells (ventral epi.) | %TdT+cells (ventral epi) | TdT+Casp3+ (ventral epi.) | Mesenchymal TdT+ | Mesenchymal  TdT+Casp3+ |
| --- | --- | --- | --- | --- | --- | --- | --- | --- |
| E9.75 | 28s | Control | 55 | 44 | 80 | n/a | 0 | 0 |
| E9.75 | 29s | Control | 51 | 37 | 73 | n/a | 0 | 0 |
| E9.75 | 27s | Control | n/a | n/a | n/a | 0 | 4 | 0 |
| E9.75 | 28s | Control | n/a | n/a | n/a | 0 | 5 | 0 |
| E9.75 | 27s | Control | 59 | 55 | 92 | n/a | 0 | 0 |
| E9.75 | 29s | Mutant | n/a | 28 | n/a | 6 | 8 | 4 |
| E9.75 | 26s | Mutant | n/a | 12 | n/a | 4 | 1 | 0 |
| E9.75 | 27s | Mutant | n/a | 23 | n/a | 0 | 2 | 0 |
| E9.75 | 27-8s | Mutant | n/a | 57 | n/a | 22 | 10 | 2 |
| E9.75 | 29s | Mutant | 82 | 18 | 23 | 11 | 1 | 1 |
| E10.5 | 32s | Control | 76 | 65 | 85 | n/a | 0 | 0 |
| E10.5 | 32s | Control | 67 | 52 | 78 | n/a | 0 | 0 |
| E10.5 | 33s | Control | 80 | 62 | 77 | 3 | 1 | 0 |
| E10.5 | 33s | Mutant | 80 | 11 | 14 | n/a | 4 | 3 |
| E10.5 | 33s | Mutant | 75 | 14 | 19 | 11 | 2 | 2 |
| E10.5 | 31s | Mutant | 78 | 15 | 19 | 9 | 0 | 0 |
| E10.5 | 34s | Mutant | 102 | 7 | 7 | 3 | 4 | 2 |
| E10.5 | 32s | Mutant | n/a | 20 | n/a | 13 | 3 | 3 |
